# Identification of LCA-binding Glycans as a Novel Biomarker for Esophageal Cancer Metastasis using a Lectin Array-based Strategy

**DOI:** 10.7150/jca.43806

**Published:** 2020-06-01

**Authors:** Min Xia, Jun Shao, Meimei Qiao, Zhiguo Luo, Xinzhou Deng, Qing Ke, Xiaoxia Dong, Li Shen

**Affiliations:** 1 Department of Clinical Oncology, Taihe Hospital, Hubei University of Medicine, Shiyan, Hubei 442000, P.R. China.; 2 Department of Biochemistry, School of Basic Medical Sciences, Hubei University of Medicine, Shiyan, Hubei 442000, P.R. China.; 3 Department of Pharmacology, School of Basic Medical Sciences, Hubei University of Medicine, Shiyan, Hubei 442000, P.R. China.; 4 Hubei Key Laboratory of Embryonic Stem Cell Research, Hubei University of Medicine, Shiyan,Hubei 442000, P.R. China.

**Keywords:** Lectin, glycan, esophageal cancer, metastasis

## Abstract

Esophageal cancer (EC) is a unique and heterogeneous disease diagnosed mostly at advanced stages. Altered glycans presented on cell surfaces are involved in the occurrence and development of malignancy. However, the effects of glycans on EC progression are largely unexplored. Here, a lectin array was utilized to detect the glycan profiling of the normal esophageal mucosal epithelial cell line and two EC cell lines. The binding of *Lens culinaris* lectin (LCA) to EC cells was found to be stronger than that of the normal cells. Lectin immunohistochemical staining revealed that LCA-binding glycans were markedly elevated in EC tissues compared to adjacent non-cancerous tissues. LCA staining was significantly associated with lymph node metastasis, depth of invasion, TNM stage and poor overall survival of EC patients. Added LCA to block LCA recognized glycans could inhibit the migration and invasion of EC cells. Further analysis revealed that blocking the biosynthesis of LCA-binding glycans by tunicamycin attenuated cellular migratory and invasive abilities. Additionally, a membrane glycoprotein CD147 was recognized as a binder of LCA. There was a positive correlation between LCA-binding glycans and CD147 expression in clinical samples. Interestingly, CD147 inhibition also reduced cell migration and invasion. These findings indicated that LCA-binding glycans may function as a novel indicator to predict metastasis for patients with EC.

## Introduction

As one of the most common malignant tumors, esophageal cancer (EC) is a unique and heterogeneous disease diagnosed mostly at advanced stages. This disease ranks seventh in terms of incidence (572,000 new cases) and sixth in mortality overall (509,000 deaths) in the world 1. For many years, EC has been a serious public health problem in China 2. EC has a high incidence of recurrence and metastasis, with a 5-year overall survival rate of < 20% 3. Although significant advances in EC treatment have been achieved by surgery, chemotherapy or radiotherapy, the prognosis for EC patients remains poor. Thus, exploring novel mechanisms and critical signaling pathways will help to the improvement of current therapeutic strategies for EC.

Glycans are highly variable and structurally diverse compounds presented on cell surfaces 4. The diversity in glycan structures enables them to participate in the occurrence and progression of various tumors 5. Aberrant glycans can serve as prominent biomarkers for cancer detection and act as effective therapeutic targets for anti-cancer drugs 6,7. Recently, increasing studies have found that the invasion and metastasis of various tumors are linked to altered cell-surface glycans. For example, changes in O-glycans were reported to be involved in the invasive behavior of oral squamous cell carcinoma 8. Artificial modification of cell-surface sialic acid modulated cell invasion in malignant lymphoma 9. Increased GlcNAc-branched N-glycan levels were closely related to metastasis of glioma, colon cancer, gastric cancer and hepatocellular carcinoma 10-12. However, the effects of glycans on EC progression are largely unexplored. In our previous study, we confirmed that inhibition of core 1 O-glycans attenuated irradiation-enhanced migration and invasion of EC cells 13. We, therefore, wanted to gain further insight into the mechanisms underlying glycans-mediated metastasis in EC.

Lectins have been the principal means of investigating cell surface glycan patterns 14. The lectin microarray is an emerging and straightforward technology for glycomic analysis 15. In the present study, we explored the differences in glycans between the normal esophageal mucosal epithelial cell line and two EC cell lines using a lectin microarray. Our data demonstrated for the first time that* Lens culinaris* lectin (LCA) specifically bound to metastasis-associated glycans in EC.

## Materials and Methods

### Cell lines and culture

Human EC cell lines (Eca-109 and KYSE-150), obtained from Procell (Wuhan, China), were maintained in DMEM (Gibco-BRL, Carlsbad, CA, USA) supplemented with 10% fetal bovine serum (FBS; HyClone, Chicago, IL, USA). Human normal esophageal mucosal epithelial cell line Het-1A, obtained from ATCC (Manassas, VA, USA), was maintained in BEGM medium kit (Lonza/Clonetics Corporation, Walkersville, MD, USA). All cells were cultured in a 5% CO_2_ atmosphere at 37 °C.

### Lectin microarray analysis

Lectin microarray (purchased from BC Biotechnology, Guangdong, China) was produced using 37 lectins with different glycan-binding specificities 16. Lectin microarray screening for cell surface glycans was performed in our lab as described previously 17. Briefly, the harvested Eca-109, KYSE-150, and Het-1A cells were washed 3 times with cold phosphate-buffered saline (PBS), marked with CFDA-SE (Life Technologies, Carlsbad, CA, USA), and probed on a lectin microarray 17. The signal intensities of greater than or equal to 3 standard deviations (SD) above background, which scanned by a GenePix 4200B scanner (Molecular Devices, Sunnyvale, CA, USA), were considered as positive signals 16,18.

### Tissue samples and immunohistochemical (IHC) staining

A total of 91 EC and 31 adjacent non-tumor tissues were acquired from patients who had undergone surgical treatment in the Taihe Hospital, Hubei University of Medicine (Hubei, China). Inclusion criteria: (1) patients were pathologically diagnosed with esophageal cancer; (2) patients had not received chemical treatment or physical therapy before surgery; (3) patients with complete clinical data. Exclusion criteria: (1) patients with other malignant tumors; (2) patients without complete clinical data. All patients consented to the use of their tissue samples. This study was approved by the Research Ethics Committee of the Hubei University of Medicine. IHC staining was carried out following a standard protocol 10. To detect the expression of LCA-binding glycans, LCA (L-1040, Vector Labs, Burlingame, CA, USA) served as the primary antibody. Horseradish peroxidase (HRP)-conjugated streptavidin (Beyotime, Jiangsu, China) was used as the secondary antibody (Beyotime). To measure CD147 expression, the samples were incubated with the primary antibody (anti-CD147, ab64616, Abcam, Cambridge, MA, USA) and detected with an HRP-labeled secondary antibody. The extent and intensity of IHC staining were measured as previously described 18. A staining index (with values from 0 to 12) was obtained as the staining intensity (negative=0, weak=1, moderate=2, or strong=3) multiplied by the proportion of immunopositive tumor cells (0-25%=1, 25-50%=2, 50-75% =3, or 75-100% =4).

### Transwell migration and invasion assays

Using a serum-free medium, the density of Eca-109 and KYSE-150 was adjusted to 2×10^5^ cells/ml. Then 200 µl of cell suspension was added to the upper chamber of an insert (8.0 µm pore size; Costar, Cambridge, MA, USA). The lower chamber was filled with medium containing 20% FBS 19. To monitor cell invasion, the upper chambers of transwell inserts were coated with matrigel. To monitor cell migration, no matrigel was coated in the upper chambers. After 24 h of incubation, cells on the underside were stained with 0.1% crystal violet (Beyotime). Images were taken at 200× magnification.

### Cell viability assay

Eca-109 and KYSE-150 cells (5×10^3^ cells/well) were seeded into 96-well plates. One day later, cells from triplicate wells were treated with the indicated different concentrations of LCA (Vector Labs), tunicamycin (Sigma, St. Louis, MO, USA), or CD147 blocking antibody (Fitzgerald, MA, USA). Following incubation for 24 h, cell viability in each group was determined using the Cell Counting Kit-8 (CCK-8; Beyotime). Absorbance at 450 nm was measured using a microplate reader (BioTek, Germany). The 50% inhibitory concentration (IC50) was calculated through the formula 20.

### Lectin blotting and western blotting analysis

RIPA lysis buffer (Beyotime) was used to extract total protein. A BCA Protein Quantitative Kit (Beyotime) was adopted to detect protein concentrations. Protein was separated by SDS-PAGE (10% gel) and transferred to PVDF membranes (Millipore, Billerica, MA, USA). For lectin blotting, the membranes were blocked with a carbo-free blocking solution (Vector Labs) and subsequently incubated with biotin-labeled LCA. For western blotting, the membranes were blocked with 5% non-fat milk. Primary antibodies (Absin, Shanghai, China) were used including anti-MMP2, anti-MMP-9, anti-p53, anti-Bcl-xL, anti-CylinD1, anti-BAX and anti-pAkt. The signal was evaluated using an enhanced chemiluminescence detection kit (Beyotime). An antibody against GAPDH (Absin) served as an endogenous reference.

### Lectin pull-down and LC-MS/MS analysis

To capture the glycoproteins, proteins from whole-cell extractions were incubated with LCA-conjugated agarose beads (Vector Labs) at 4°C overnight. After washing three times with PBS, samples were separated by SDS-PAGE on a 10% gel and stained with coomassie blue R250 (Beyotime). In-gel trypsin digestion and LC-MS/MS analysis were done as previously reported 21.

### Statistical analysis

All the values obtained from at least three independent experiments were analyzed using GraphPad Prism 5.0 (La Jolla, CA, USA). Data were expressed as mean ± SD. Comparisons were conducted by student's t-test or one-way analysis of variance (ANOVA). Kaplan-Meier method was applied to determine overall survival rates. P < 0.05 was considered to be significant.

## Results

### Identification of metastasis-associated glycans on EC cell lines

**Figure 1:**
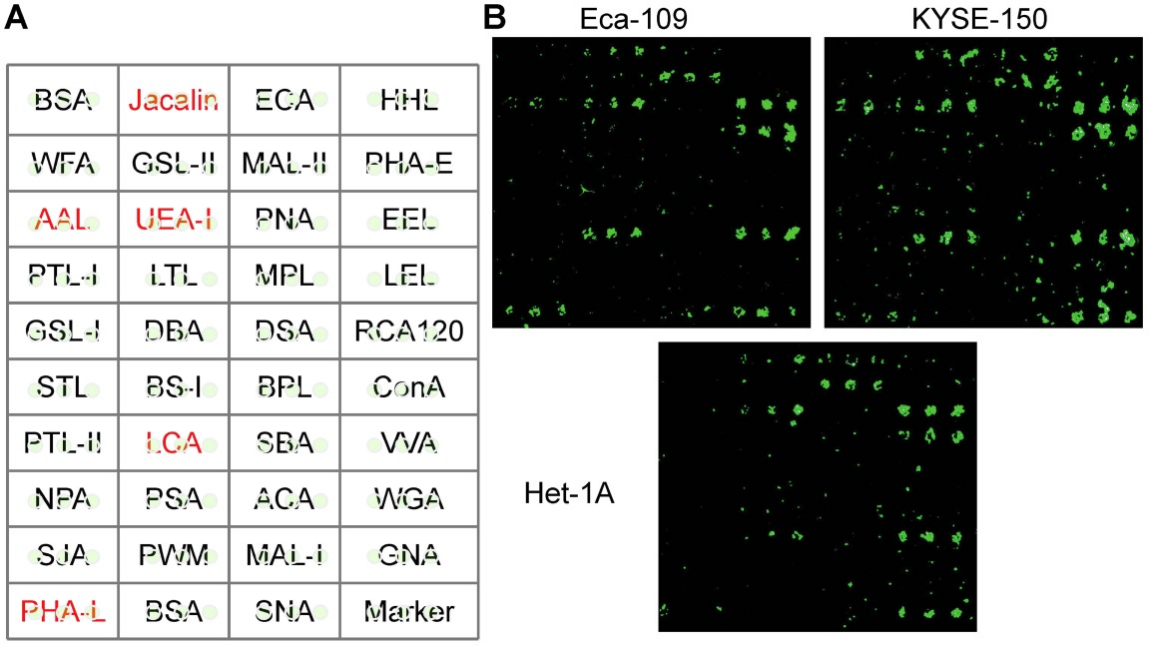
** Identification of metastasis‑specific lectin binding to EC cells.** (A) Layout of the lectin microarray containing 37 lectins. (B) Representative binding patterns of Het-1A, Eca-109 and KYSE-150 cell lines.

To identify which glycans appear and how cancer cells differ from normal cells, Het-1A, Eca-109, KYSE-150 cell lines were chosen for this study. The differences in glycans were compared by a lectin microarray, in which each lectin was present in triplicate (Fig. 1A). The specific glycans recognized by the lectins and the relative fold change of each lectin were summarized in Table 1. We found that lectins Jacalin, LCA, AAL, PHA-L, and UEA-I exhibited stronger binding affinities to cancer cells, as compared to normal cells (Fig. 1B). The most significant difference was seen for LCA. It has been reported that cancer cells frequently possess higher metastatic properties than normal cells 22,23. Based on this information, we speculated that LCA-binding glycans played an important role in EC metastasis. We, therefore, selected it for further analysis.

### Validation of metastasis-specific LCA-binding patterns in EC patients

**Figure 2:**
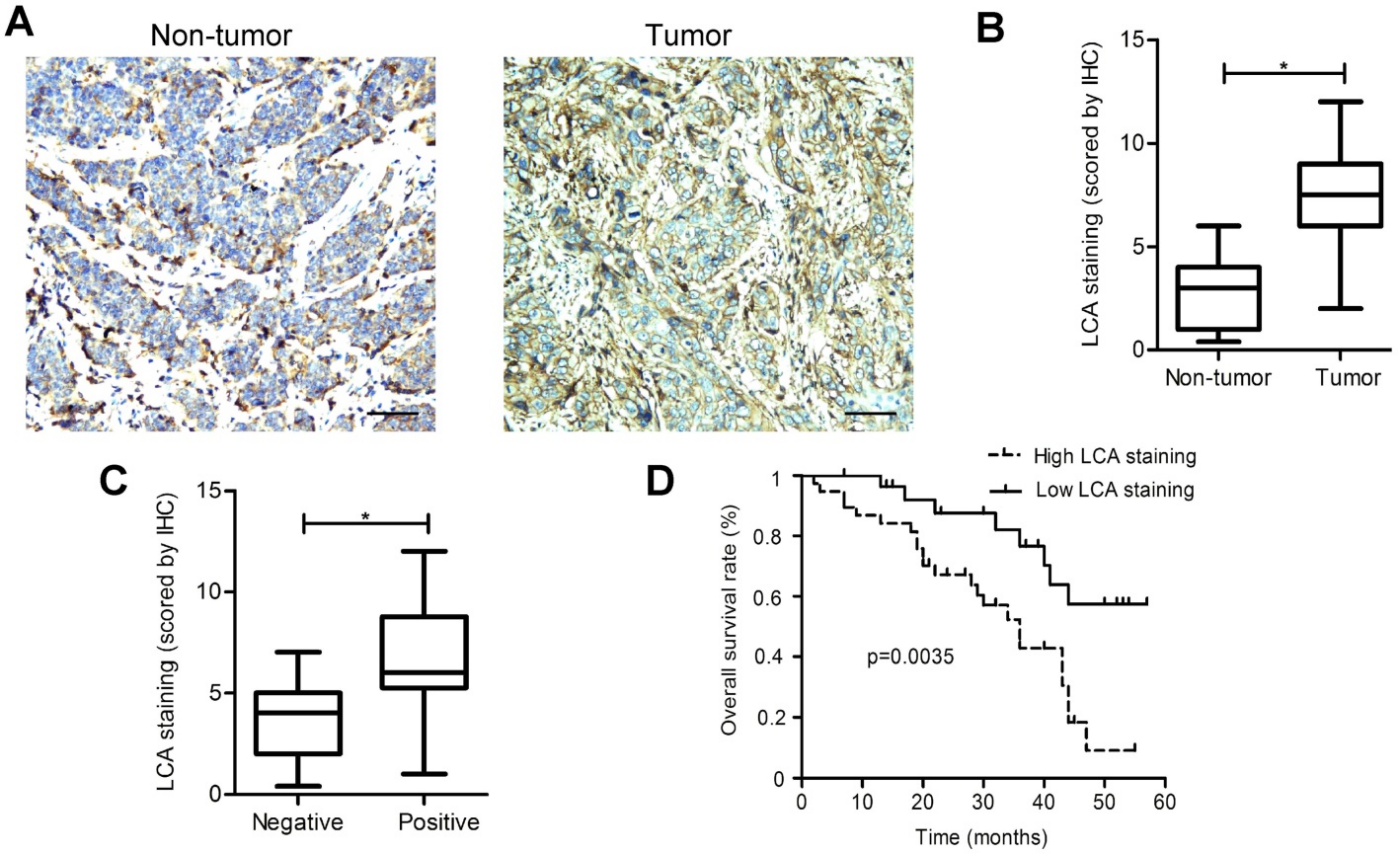
** IHC staining for LCA-binding glycans.** (A) Representative photomicrographs for LCA-binding glycans in EC and adjacent non-tumor tissues. (B) Analysis of LCA-binding glycans based on the scoring data. (C) Comparison of LCA staining intensities in the metastasis-positive group and metastasis-negative group. (D) Kaplan-Meier survival curves for LCA staining. Scale bar represents 100 µm. * P < 0.05.

LCA staining was found to be significantly elevated in EC tissues as compared to adjacent non-tumor tissues (P < 0.05; Fig. 2A-B). A significant association between the positive staining of LCA and the occurrence of lymph node metastasis, depth of invasion, and TNM stage was observed in patients (Table 2). However, no significant differences were detected between the binding tendency of LCA and gender, age, tumor size, or differentiation. Among EC tissues, LCA staining intensities in the metastasis-positive group were markedly higher than those in the metastasis-negative group (P < 0.05; Fig. 2C). Kaplan-Meier survival analysis illustrated that, compared with those showing low LCA staining; patients with high LCA staining had shorter overall survival (P < 0.05; Fig.2D). Our results strongly suggested that the presence of LCA-specific glycans could be used as a useful biomarker of metastatic potential in EC.

### Added LCA to block LCA-binding glycans inhibits migration and invasion of EC cells

**Figure 3:**
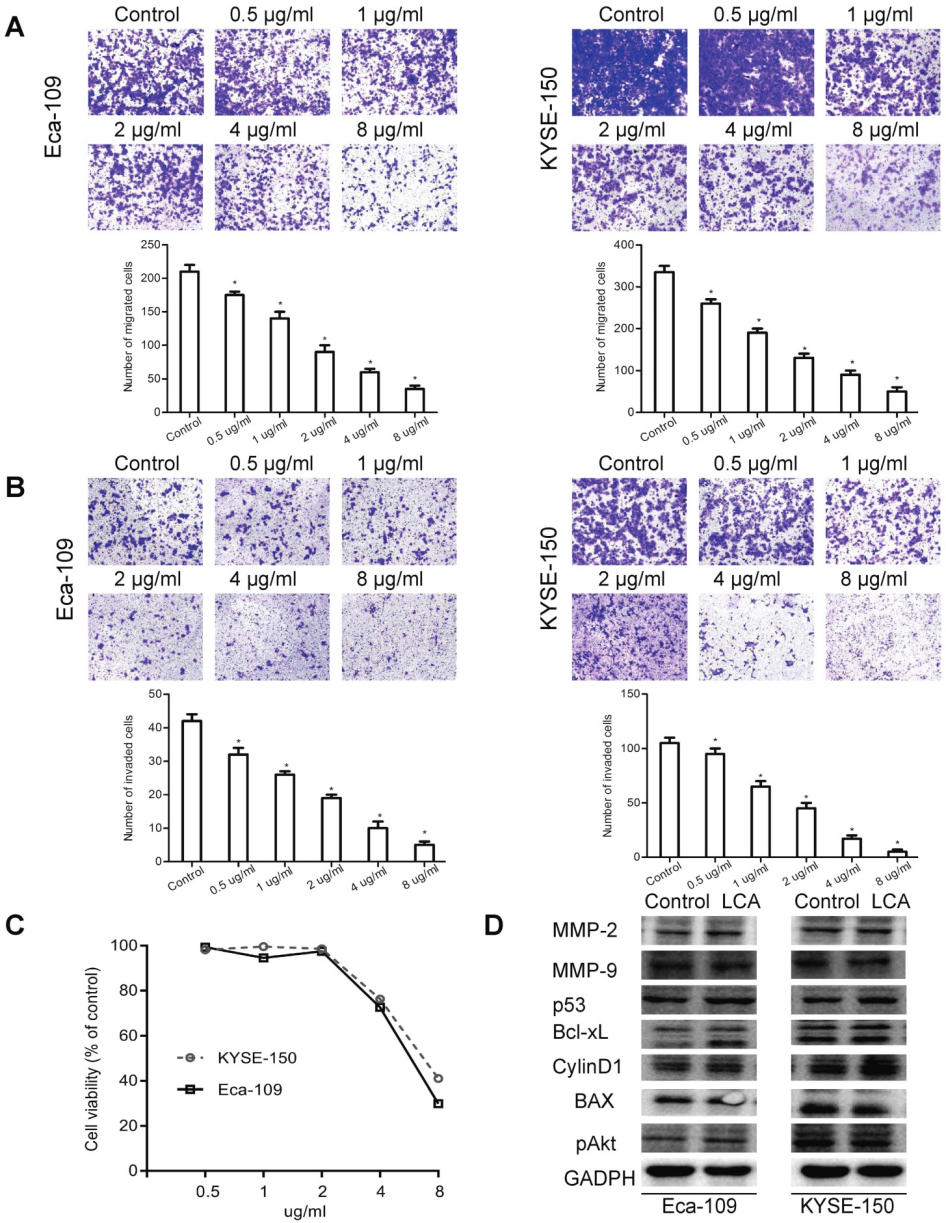
** Effects of LCA on EC cell migration and invasion.** (A) Representative images and the mean migration number for each treatment group. (B) Representative images and the mean invasion number for each treatment group. (C) Cell viability for each treatment group. (D) Expression levels of the MMPs and PI3K/Akt pathway-associated proteins for cells treated with LCA at the IC50 concentration. Untreated cells served as a control. * P < 0.05 compared to the control group.

To explore whether lectin LCA affected any processes associated with metastasis, migration and invasion were studied by transwell assays. We observed that the migration of Eca-109 and KYSE-150 cells was suppressed by LCA in a dose-dependent manner, particularly at a higher concentration (Fig. 3A). Compared with the control group, LCA dose-dependently reduced the number of invaded Eca-109 and KYSE-150 cells (Fig. 3B).

To investigate the mechanisms underlying the anti-metastatic effects of LCA, cells were firstly incubated with different concentrations of LCA. As shown in Fig. 3C, cell viability decreased linearly with increasing LCA concentrations. Correspondingly, the IC50 values of Eca-109 and KYSE-150 were 6µg/ml and 7 µg/ml. Then cells were treated with LCA at the IC50 concentration. It was reported that the matrix metalloproteinases (MMPs) and PI3K/Akt signaling pathway-related proteins (p53, CylinD1, Bcl-2, Bax, and pAkt) regulated the invasion and metastasis in many types of cancer cells 24-26. Thus, the expression of related molecules was analyzed by western blotting. We found that the levels of MMP-2, MMP-9, p53, Bcl-xL, CylinD1, BAX, and pAkt were not changed after LCA treatment in Eca-109 and KYSE-150 cells (Fig. 3D). Taken together**,** these findings provided evidence that added LCA to block LCA-binding glycans inhibited cell migration and invasion. These effects of LCA may not be related to the expression of MMPs and the PI3K/Akt pathway.

### Blocking the synthesis of LCA-binding glycans by tunicamycin attenuates the migratory and invasive abilities of EC cells

**Figure 4:**
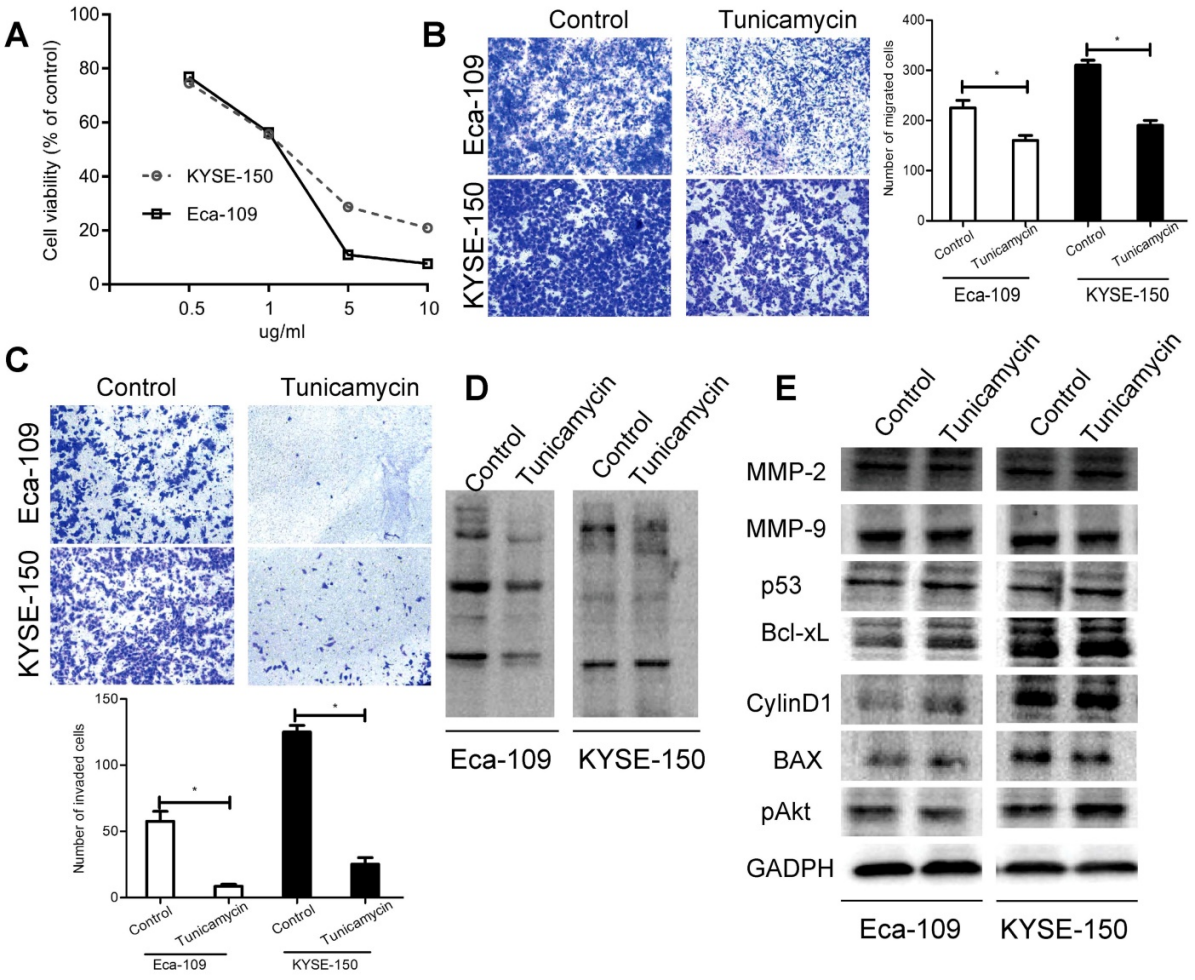
** Effects of tunicamycin on EC cell migration and invasion.** (A) Cell viability for each treatment group. (B) Representative images and the mean migration number for cells treated with tunicamycin at the IC50 concentration. (C) Representative images and the mean invasion number for cells treated with tunicamycin at the IC50 concentration. (D) Expression levels of LCA-binding glycans for cells treated with tunicamycin at the IC50 concentration. (E) Expression levels of the MMPs and PI3K/Akt pathway-associated proteins for cells treated with tunicamycin at the IC50 concentration. Cells treated with DMSO served as a control. * P < 0.05 compared to the control group.

Tunicamycin is known to be an inhibitor of the synthesis of N-linked glycans 27. Results of CCK-8 assay showed that tunicamycin induced anti-proliferative activity, displaying an IC_50_ of 1 µg/ml in both cell lines (Fig. 4A). We also found that tunicamycin at the IC50 concentration decreased the migration and invasion of Eca-109 and KYSE-150 cells (Fig. 4B-C). Besides, lectin blotting analysis confirmed that tunicamycin caused a suppressive effect on the expression of LCA-binding glycans (Fig. 4D). However, tunicamycin had no obvious effects on the levels of MMP-2, MMP-9, p53, Bcl-xL, CylinD1, BAX and pAkt (Fig. 4E). Our results further confirmed a modulating influence of LCA-binding glycans on cell metastasis.

### Identification of CD147 as an LCA-recognized membrane glycoprotein

**Figure 5:**
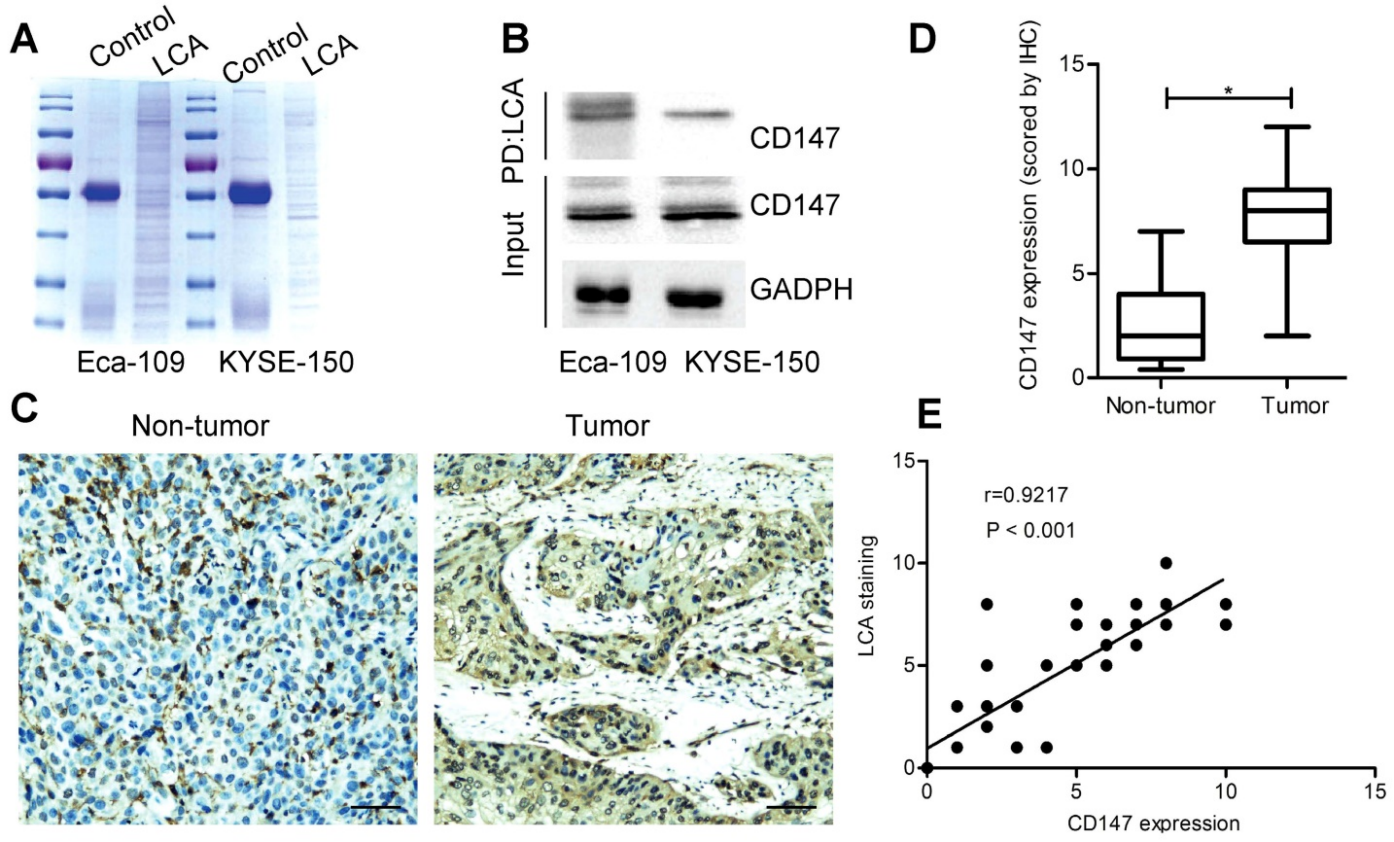
** Identification of CD147 as an LCA-recognized glycoprotein.** (A) Coomassie blue staining for immunoprecipitated samples. (B) LCA immunoprecipitation assay for Eca-109 and KYSE-150 cells. PD: pull down. (C) Representative photomicrographs for CD147 expression in EC and adjacent non-tumor tissues. Scale bar represents 100 µm. (D) Analysis of CD147 expression based on the scoring data for IHC (E) Correlation between LCA-binding glycans and CD147 expression in EC tissues. IgG served as a control. P < 0.05.

To identify potential glycoproteins recognized by LCA, lectin pull-down assay was performed (Fig. 5A). As a result, a membrane glycoprotein CD147 was detected both in Eca-109 and KYSE-150 cells (Table S1). LCA immuno-precipitation revealed that CD147 was efficiently co-precipitated with LCA (Fig. 5B). Then we carried out IHC staining and measured CD147 expression in specimens. The results showed that the expression of CD147 in EC tissues was higher than that in adjacent non-tumor tissues (P < 0.05; Fig. 5C-D). CD147 expression was considerably correlated with lymph node metastasis, depth of invasion, and TNM stage (P < 0.05; Table 2). Based on each paired IHC score, there was a positive correlation between LCA-binding glycans and CD147 (P < 0.001; Fig. 5E).

### Inhibition of CD147 reduces cell migration and invasion

**Figure 6:**
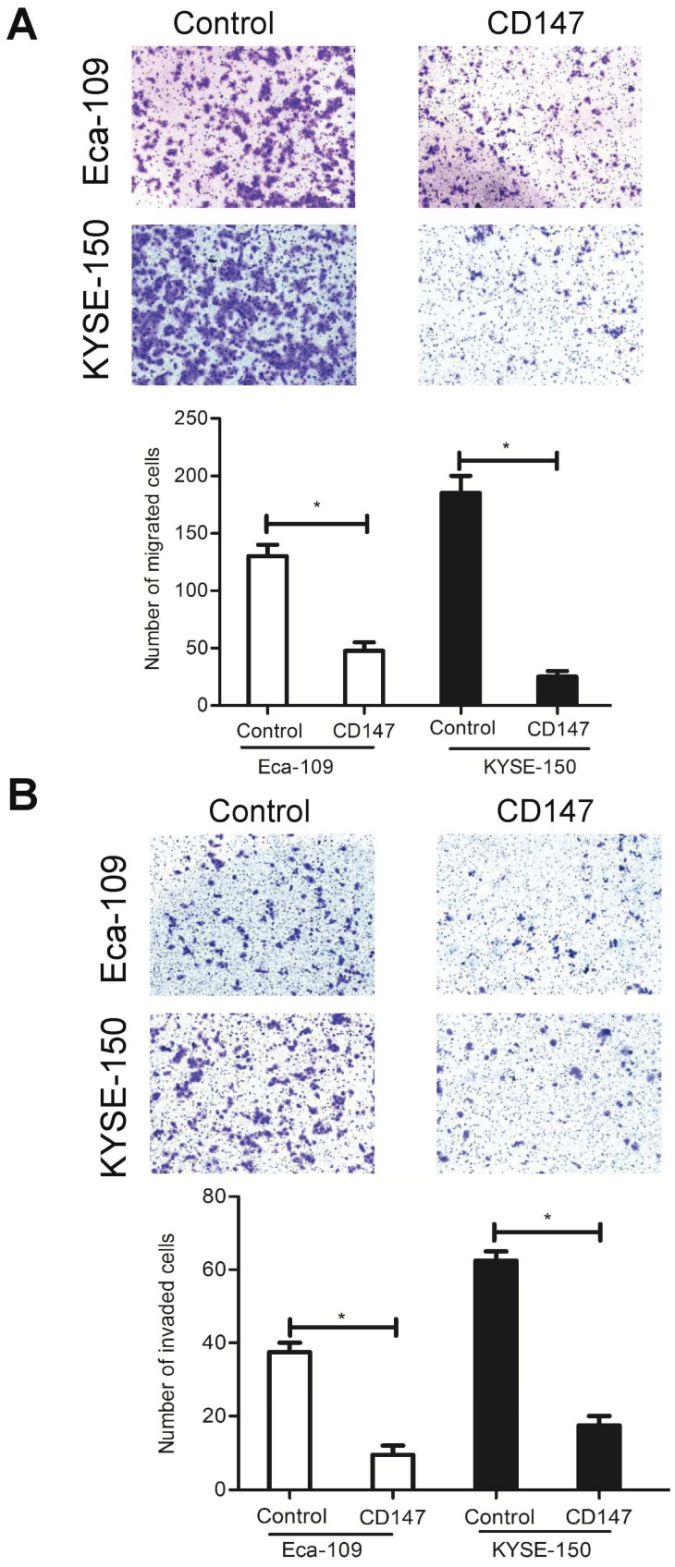
** Effects of CD147 on EC cell migration and invasion.** Eca-109 and KYSE-150 cells were pre-treated with 1 µg/ml and 2 µg/ml CD147 blocking antibody, respectively. (A) Representative images and the mean migration number for each group. (B) Representative images and the mean invasion number for each group. IgG served as a control. * P < 0.05 compared to the control group.

As described above, LCA-binding glycans had a strong impact on cell migration and invasion. To determine whether these effects were mediated by the LCA-CD147 interaction, EC cells were cultured in the presence of CD147 function-blocking antibody at a lower concentration. We found that the antibody treatment decreased the migration ability and the invasiveness of Eca-109 and KYSE-150 cells (Fig. 6A-B). These data suggested that LCA-binding glycans regulated cell metastasis, at least in part, via modulation of CD147.

## Discussion

Metastasis is a major contributor to the mortality of cancer patients 28. This process is associated with cancer cell extravasation and subsequent invasion of normal surrounding tissue, which is mediated by cell-surface mechanisms 16. Cell surface-associated glycans regulate many cellular processes, including adhesion, migration, signaling, and extracellular matrix organization 29. Growing evidence has shown that aberrantly expressed glycans recognized by lectins can serve as important regulators for cancer metastasis 8,12,18,30. In the current study, we demonstrated for the first time that lectin LCA specifically bound to metastasis-associated cell-surface glycans in EC. The expression of LCA-binding glycans was positively correlated with lymph node metastasis, depth of invasion, TNM stage and poor overall survival of patients. We also confirmed that inhibition of LCA-binding glycans could suppress EC cell migration and invasion. Our findings provided a novel insight into the mechanisms responsible for EC metastasis.

Several molecules and pathways have been proven to contribute to EC cell migration and invasion. For example, amplified or overexpressed MMP-2 and MMP-9 were associated with the invasive and metastatic properties of EC cells 31,32. The PI3K/Akt pathway was an important signaling pathway that modulated cell migration and invasion in EC 7, 33. However, whether LCA-binding glycans participate in EC metastasis mediated by the MMPs and PI3K/Akt pathway is still unknown. In this study, after examining the inhibition effects of LCA and tunicamycin on migration and invasion, we then investigated the levels of MMPs and the PI3K/Akt signaling pathway-related proteins. Western blotting showed that LCA and tunicamycin had no obvious effects on the levels of MMP-2, MMP-9, p53, Bcl-xL, CylinD1, BAX and pAkt. Therefore, blocking the biosynthesis of LCA-binding glycans inhibited EC cell migration and invasion maybe not via the MMPs and PI3K/Akt signaling pathway.

CD147, also known as EMMPRIN, Basign, and HAb18G, is a widely expressed cell surface protein with multiple glycosylated forms. Previous studies have shown that lectins PHA-L, PHA-E, and LEL could directly bind to CD147 in many types of cancer cells 12,34,35. In the present study, we identified CD147 as a lectin LCA-recognized membrane glycoprotein in EC cells. Recently, the functional roles of CD147 in cancer proliferation, migration, and invasion have been extensively reported. For instance, inhibition of CD147 expression decreased proliferation and invasion in colorectal cancer stem cells 36. *In vitro* and *in vivo* prostate cancer metastasis can be modulated by CD147 37. CD147 was a key regulator of metastasis in non-small cell lung cancer 19, bladder cancer 38, EC 39 and gastric cancer 40. Our findings agree with these reports that CD147 was upregulated in EC tissues. Moreover, the antibody blockade of CD147 reduced cell migration and invasion. Thus, preventing the binding of CD147 to LCA-specific glycans by CD147 blocking antibody was also involved in EC metastasis.

In summary, lectin LCA specifically bound to metastasis-associated cell-surface glycans in EC. Additionally, a membrane glycoprotein CD147 was recognized as a binder of LCA, which played a critical role in mediating these effects. Therefore, LCA-binding glycans may function as a novel indicator to predict metastasis for patients with EC.

## Supplementary Material

Supplementary table S1.

## Figures and Tables

**Table 1:** The relative fold change of each lectin in Eca-109 and KYSE-150 cells

Lectin	Origin	Preferred glycan structures	Compared with Het-1A (Fold change)	
			Eca-109/Het-1A	KYSE-150/Het-1A
Jacalin	*Artocarpus integrifolia*	Galβ1-3GalNAcα-Ser/Thr(T) and GalNACα-Ser/Thr(Tn)	1.88*	2.54*
ECA	*Erythrina cristagalli*	Galβ-1,4GlcNAc	0.74	1.34
HHL	*Hippeastrum hybrid*	Polymannose (α-1,3) and (α-1,6) linked mannose	-	-
WFA	*Wisteria floribunda*	GalNAcα/β-1,3/6Gal	-	-
GSL-II	*Griffonia simplicifolia*	GlcNAc	-	-
MAL-II	*Maackia amurensis*	Sia2-3Galβ1-4Glc (NAc)	1.14	1.09
PHA-E	*Phaseolus vulgaris*	Bisecting GlcNAc and biantennary N-glycans	-	-
PTL-I	*Psophocarpus tetragonolobus*	αGalNAc	-	-
SJA	*Sophora japonica*	Terminal in GalNAc and Gal	-	-
PNA	*Arachis hypogaea*	Galβ1-3GalNAcα-Ser/Thr(T)	-	-
EEL	*Euonymus europaeus*	Galα1-3(Fucα1-2)Gal	1.03	1.17
AAL	*Aleuria aurantia*	Fucα-1,6GlcNAc, Fucα-1,3LacNAc	1.97*	2.41*
LTL	*Lotus tetragonolobus*	α-L-Fuc	-	-
MPL	*Maclura pomifera*	αGalNAc	-	-
LEL	*Lycopersicon esculentum*	Poly-LacNAc and (GlcNAc)n	1.29	1.36
GSL-I	*Griffonia simplicifolia*	αGalNAc, αGal	-	-
DBA	*Dolichos biflorus*	GalNAcα-Ser/Thr(Tn)	-	-
LCA	*Lens culinaris*	α-Man and Fucα-1,6GlcNAc (core fucose)	2.75*	3.44*
RCA120	*Ricinus communis*	Gal and GalNAc	-	-
STL	*Solanum tuberosum*	(GlcNAc)n	-	-
BS-I	*Bandeiraea simplicifolia*	αGal and αGalNAc	-	-
ConA	*Canavalia ensiformis*	α-Man (inhibited by presence of bisecting GlcNAc)	-	-
PTL-II	*Psophocarpus tetragonolobus*	Gal	-	-
DSA	*Datura stramonium*	β1-4GlcNAc and LacNAc	-	-
SBA	*Glycine max*	Terminal GalNAc (especially GalNAcα1-3Gal)	-	-
VVA	*Vicia villosa*	GalNAc and GalNAcα-Ser/Thr(Tn)	1.37	1.49
NPA	*Narcissus pseudonarcissus*	Polymannose(α-1,6)linked mannose	-	-
PSA	*Pisum sativum*	Fucα-N-acetylchitobiose -man	-	-
ACA	*Amaranthus caudatus*	Galβ1-3GalNAcα-Ser/Thr(Tn)	-	-
WGA	*Triticum unlgaris*	Terminal GlcNAc and (GlcNAc)n	-	-
UEA-I	*Ulex europaeus*	Fucoseα1-2Galβ1-4Glc(NAc)	1.74*	2.33*
PWM	*Phytolacca americana*	GlcNAc	-	-
MAL-I	*Maackia amurensis*	Galβ-1,4GlcNAc and Sia2-3Galβ1-4Glc(NAc)	-	-
GNA	*Galanthus nivalis*	(α-1,3)Man	-	-
BPL	*Bauhinia purpurea alba*	Galβ1-3GalNAc	-	-
PHA-L	*Phaseolus vulgaris*	Tri/Tetra-antennary complex-type N-glycan	2.61*	2.21*
SNA	*Sambucus nigra*	Sia2-6Galβ1-4Glc(NAc)	1.01	-

Signal intensities obtained from lectin microarrays were normalized. *P < 0.05; -: Negative signal.

**Table 2:** Clinicopathological features of esophageal cancer patients

Variables	Number	LCA staining		*P*-value	CD147 expression		*P*-value
		Low (n=39)	High (n=52)		Low (n=31)	High (n=60)	
**Age**							
<60	40	20	20	0.399	21	19	0.603
≥60	51	19	32		10	41	
**Gender**							
male	43	15	28	0.621	22	21	0.317
female	48	24	24		9	39	
**Size of tumor**							
<5 cm	44	22	22	0.134	13	31	0.281
≥5 cm	47	17	30		18	29	
**Differentiation**							
Moderate-High	50	25	25	0.129	10	40	0.275
Low	41	14	27		21	20	
**Depth of invasion**							
T1+T2	36	29	7	0.004*	20	16	0.013*
T3+T4	55	10	45		11	44	
**Lymph node metastasis**							
Positive	61	14	47	0.001*	11	50	0.002*
Negative	30	25	5		20	10	
**TNM stage**							
I+II	45	27	18	0.015*	24	21	0.016*
III +IV	46	12	34		7	39	

*P < 0.05.
